# Facile Synthesis of Ni-MgO/CNT Nanocomposite for Hydrogen Evolution Reaction

**DOI:** 10.3390/nano14030280

**Published:** 2024-01-29

**Authors:** Panneerselvam Mohana, Melkiyur Isacfranklin, Rathinam Yuvakkumar, Ganesan Ravi, Lakshmanan Kungumadevi, Sundaramoorthy Arunmetha, Jun Hyun Han, Sun Ig Hong

**Affiliations:** 1 Department of Physics, Alagappa University, Karaikudi 630003, Indiaisacfranklin3@gmail.com (M.I.); ravig@alagappauniversity.ac.in (G.R.); 2 Department of Physics, Chandigarh University, Mohali 140413, India; 3 Department of Physics, Mother Terasa Women’s University, Kodaikanal 624101, India; sivarivudevi@gmail.com; 4 Department of Electronics and Communication Engineering, Koneru Lakshmaiah Education Foundation, Guntur 522502, India; sarunmetha@kluniversity.in; 5 Department of Materials Science and Engineering, Chungnam National University, Daejeon 34134, Republic of Korea; jhhan@cnu.ac.kr

**Keywords:** water splitting, hydrogen evolution reaction, chemical vapor deposition, overpotential, hydrogen production

## Abstract

In this study, the pristine MgO, MgO/CNT and Ni-MgO/CNT nanocomposites were processed using the impregnation and chemical vapor deposition methods and analyzed for hydrogen evolution reaction (HER) using the electrochemical water splitting process. Furthermore, the effect of nickel on the deposited carbon was systematically elaborated in this study. The highly conductive carbon nanotubes (CNTs) deposited on the metal surface of the Ni-MgO nanocomposite heterostructure provides a robust stability and superior electrocatalytic activity. The optimized Ni-MgO/CNT nanocomposite exhibited hierarchical, helical-shaped carbon nanotubes adorned on the surface of the Ni-MgO flakes, forming a hybrid metal–carbon network structure. The catalytic HER was carried out in a 1M alkaline KOH electrolyte, and the optimized Ni-MgO/CNT nanocomposite achieved a low (117 mV) overpotential value (ɳ) at 10 mA cm^−2^ and needed a low (116 mV/dec) Tafel value, denotes the Volmer–Heyrovsky pathway. Also, the high electrochemical active surface area (ECSA) value of the Ni-MgO/CNT nanocomposite attained 515 cm^2^, which is favorable for the generation of abundant electroactive species, and the prepared electrocatalyst durability was also performed using a chronoamperometry test for the prolonged duration of 20 h at 10 mA cm^−2^ and exhibited good stability, with a 72% retention. Hence, the obtained results demonstrate that the optimized Ni-MgO/CNT nanocomposite is a highly active and cost-effective electrocatalyst for hydrogen energy production.

## 1. Introduction

In an ever-expanding global landscape, the imperative for energy harvesting becomes increasingly vital to minimize energy loss and gather many different forms of energies to meet the escalating demand for energy driven by a surging global population. This necessity is further featured by the imperative to sustain socioeconomic progress, enhance well-being and safeguard public health. Simultaneously, the exponential growth in the population has become a catalyst for a spectrum of environmental challenges, notably the alarming rise in greenhouse gas emissions. Consequently, amidst this complex scenario, the pressing need arises for a transition towards renewable energy including solar, wind power, biomass and biogas, which may offer a sustainable alternative to fulfill the energy requirements of the burgeoning population while mitigating the adverse impacts associated with the depletion of finite fossil resources. This shift towards renewable energy not only addresses the immediate energy needs, but also aligns with the imperative of fostering a more sustainable environment and contributing to a more environmentally conscious energy landscape [1]. In the pursuit of establishing a clean and sustainable energy production paradigm, molecular hydrogen (H_2_) has garnered recognition as a carbon-free alternative. Renowned for its exceptional gravimetric energy density, reaching approximately 282 KJ mol^−1^, hydrogen stands out as a highly efficient and eco-friendly option. This characteristic makes it an appealing choice in the quest for sustainable energy solutions, offering the potential to significantly reduce carbon emissions [2]. Industrial hydrogen production, so far, has predominantly relied on the conventional steam–methane reforming method. A notable drawback of this approach, however, is the inherent generation of hazardous carbon dioxide (CO_2_) emissions during the reaction process. This poses a significant environmental concern, as it contributes to the ever-increasing issue of greenhouse gas emissions, underscoring the pressing need for alternative and sustainable methods in hydrogen production to mitigate these adverse environmental impacts [3]. Over the past few decades, electrocatalytic water splitting was adopted by researchers as an effective technique for pure hydrogen production [4,5,6,7]. The electrocatalysis of water primarily consists of two half redox reactions: anodic oxygen evolution (OER), with a four-electron step transfer and cathodic hydrogen evolution reaction (HER), with a two-electron step transfer [5]. The efficient hydrogen evolution reaction mainly comprises two reaction mechanisms named as the Volmer–Heyrovsky and Volmer–Tafel mechanisms. In those two steps, hydrogen generation proceeded depending on the process of adsorption (Volmer) and the desorption and recombination (Heyrovsky and Tafel) process of the adsorbed hydrogen (H*) and hydroxide ions (OH^−^), which plays a vital role in H_2_ generation and in high HER catalytic performance. To this date, noble catalysts such as Pt/C- or Ru/IrO_2_-based materials have exhibited outstanding electrocatalytic performances towards overall electrochemical water splitting [6,7,8,9,10]. Consequently, the expensive costs and instability of these worthy candidates severely limit their growth in industrial applications. Therefore, switching to efficient non-noble catalysts at a lower cost with an enhanced electrocatalytic performance is highly advisable to provide sustainable alternative energy [10,11]. Recently, transition metal-based electrocatalysts like oxides/hydroxides, sulfides, carbides, nitrides phosphides, etc., are widely explored in many energy conversion and storage applications, owing to their high stability, affordability and earth-rich nature [12]. Among many metal-oxide-based electrocatalysts, magnesium oxide (MgO) has attracted great interest because of its large surface sites, lattice defects, ion vacancies (that can promote the surface chemical kinetics) and its catalytic behavior [13,14].

The single metal oxides, however, are known to possess a lack of stability, low conductivity and inferior catalytic activity, which may obstruct the improvement of catalytic activity at the large-scale level. On the other hand, carbon materials can be used as potential additives for nanocomposite formation with the improved catalytic conductivity and active site dispersibility [15]. Carbon nanotubes have attracted significant attention in the development of advanced sustainable energy storage applications. The formation of nanocomposites of metal oxides and CNTs facilitates the access to metal carbon surfaces for better charge storage activities of high electrolyte ions and amplifies the specific material surface area [16]. Ni has been recognized as an effective element to form the metal–metal oxide carbon lattice structure due to its high redox active sites and being known to enhance electrical conductivity and stability [17]. Liu et al. [18] achieved low (70 mV) overpotential values at 10 mA cm^−2^ in an alkaline medium using Ni–Mg–La for HER studies. Darb and et al. [19] synthesized a Ni-CNT nanocomposite using the electrodeposition method and achieved low overpotential values of 82, 116 and 207 mV at 10, 20 and 100 mA cm^−2^.

So far, various synthesis methods were explored such as hydrothermal processes, microwave-assisted synthesis, electrodeposition, chemical vapor deposition, sol–gel techniques, etc., dwere used to develop the three-dimensional hierarchical nanostructures comprising metal–carbon nanocomposites with enhanced electrocatalytic activities [20,21,22,23,24]. Furthermore, the chemical vapor deposition method (CVD) is recognized as a cost-effective approach for synthesizing carbon nanotubes (CNTs). By carefully adjusting the temperature within the range of 550–1000 °C, the fast growth of CNTs along with a desired alignment of the tubular structure was obtained [25]. In the present study, the impregnation process followed by the chemical vapor deposition method was used. We also specifically focused on elucidating the heterogeneous electrocatalytic action of the composite structure in facilitating the hydrogen evolution reaction. 

## 2. Materials and Methods

### 2.1. Materials

Magnesium oxide light LR (MgO) was acquired from S.D. Fine Chem. Ltd., Mumbai, Maharashtra, India while nickel(II) nitrate hexahydrate (Ni(NO_3_)_2_·6H_2_O) was obtained from Sigma Aldrich, St. Louis, MO, USA. High-purity nitrogen (99.999%), hydrogen (99.99%) and acetylene (99.9999%) were sourced from Success Trader, Karaikudi for use in our experimental procedures.

### 2.2. Synthesis Procedure

**Scheme 1:**
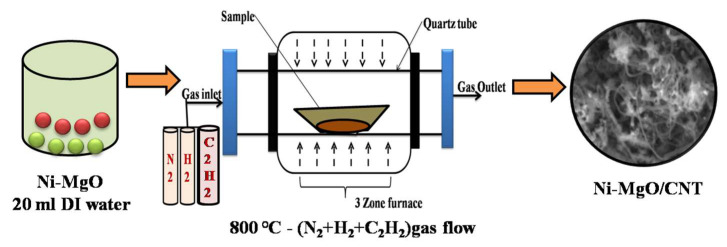
Ni-MgO/CNT nanocomposite preparation.

MgO (0.7 g) powders were placed in a CVD reactor under an inert gas atmosphere (N_2_) for 1 h 40 min to reach 500 °C, and then, a mixture of H_2_+N_2_ served as the pretreated carrier gas that assists in restructuring the catalyst and aids in transporting the hydrocarbons during the CVD process [26], with the gradual increase in temperature from 500 °C to 800 °C during the period of 1 h. After the temperature reached 800 °C, a precursor gas (C_2_H_2_) was introduced to induce slow cooling for 30 min. Meanwhile, a solution of 0.2 g Ni(NO_3_)_2_·6H_2_Odissolved in 20 mL of distilled water (stirred for 30 min) was prepared. As 0.2 g of magnesium oxide (MgO) was completely dissolved in the mixed solution after approximately 1 h, the color of the solution gradually changed from pale white to pale green. The prepared material was filtered using distilled water, acetone and methanol. Ni-MgO powder was placed in a CVD reactor under an inert gas atmosphere (N_2_) for 1 h 40 min until the temperature reached 500 °C. Then, H_2_+N_2_gases were supplied to reduce the catalyst and promote carbon nanotube growth on it for 1 h at 500 °C. The temperature was then increased from 500 to 800 °C for 1 h. After the temperature reaches 800 °C, a precursor gas(C_2_H_2_) was introduced for 30min, and subsequently, it was slowly cooled. The preparation of the Ni-MgO/CNT nanocomposite is shown in Scheme 1.

### 2.3. Characterizations

#### 2.3.1. Electrode Fabrication

The electrochemical study was conducted employing the Bio-logic SP-150 workstation was purchased from Seyssinet Pariset, France. A slurry of the working substance was carefully produced employing a MgO, MgO/CNT and Ni-MgO/CNT catalyst, activated carbon and polyvinylidene fluoride (PVDF) in 85:10:5 ratios, respectively. Manual mixing was performed in a few drops of N-methyl pyrrolidinone (NMP). Also, 3 mg of catalyst was loaded to each electrode for the electrochemical investigations. Ni substrate was subjected to precleaning with 1 M HCl in 100 mL of deionized (DI) water employing ultrasonication for 30 min, effectively removing the NiOx surface layer. For the electrochemical studies, the catalyst-coated Ni foam (1 × 1 cm^2^) served as the working electrode, Pt wire as the counter electrode and the Ag/AgCl electrode functioned as the reference electrode in a 3-electrode system specifically for HER applications. 

#### 2.3.2. Electrochemical Analysis Parameters

A total of 1 M KOH was employed as an alkaline electrolyte. All potential applied in the cyclic voltammetry analysis was referenced to the reversible hydrogen electrode (RHE) employing the Nernst equation E_RHE_ = E_Ag/AgCl_ + 0.197 + (0.059 × pH), where E_RHE_ refers to RHE potential conversion, E_Ag/AgCl_ denotes the potential of the reference electrode (Ag/AgCl), the value 0.197 represents the standard potential of the reference electrode and the pH of the KOH alkaline electrolyte was estimated as 13.6. Furthermore, double-layer capacitance (C_dl_) values were evaluated by using the formula of |j_a_ − j_c_|/2ν, where |j_a_ − j_c_| represents the difference between anodic and cathodic current densities and ν denotes the applied scan rates (mV/s). The ECSA was calculated approximately employing a formula that involved dividing the acquired double-layer capacitance value (C_dl_) by the specific capacitance of the substrate used (C_s_)ECSA(cm^2^) = C_dl_/C_s_ [27]. Also, the value 0.040 mF cm^−2^ for C_s_ was adopted, consistent with prior reports on HER activity [28]. The LSV analysis of the prepared electrode was performed at 0 to −0.6 V vs. the RHE at 2 mV/s. From the analysis, the overpotential values were evaluated at a minimum current density of 10 mA/cm^2^ using the condition ɳ = E_RHE_ − 0 V. To probe the dynamics of the electrochemical reaction process, electrochemical impedance spectroscopy (EIS)was carried out from 100 kHz to 0.1 Hz at an amplitude range of 5 mV, additionally with a chronoamperometry study, which was carried out at10 mA cm^−2^ to further evaluate the electrochemical performance. 

#### 2.3.3. Material Characterization

The structural and phase purity was studied employing a high-temperature powder X-ray diffractometer (HT-XRD) equipped with an HTK 1200N—Bruker D8 Advance, Leipzig, Germany. Additionally, small changes in the structural morphology and disorder of the carbon nanomaterials were detected using micro–laser Raman instruments (Seiki, Japan). Furthermore, the morphologies of the sample were examined through the utilization of a CAREL ZEISS EVO 18, Oberkochen, Germany scanning electron microscope and a JEOL-2100+ high-resolution transmission electron microscope, Tokyo, Japan. The composition and chemical state of the Ni-MgO/CNT were examined employing a PHI-VERSAPROBE III—X-ray photoelectron spectrometer, (ULVAC, Chigasaki, Japan). An electrochemical study was conducted employing a Biologic SP-150 instrument, Seyssinet-Pariset, France to investigate the electrochemical properties of the system under study.

## 3. Results and Discussion

### 3.1. Phases and Chemical Structure Analysis

**Figure 1:**
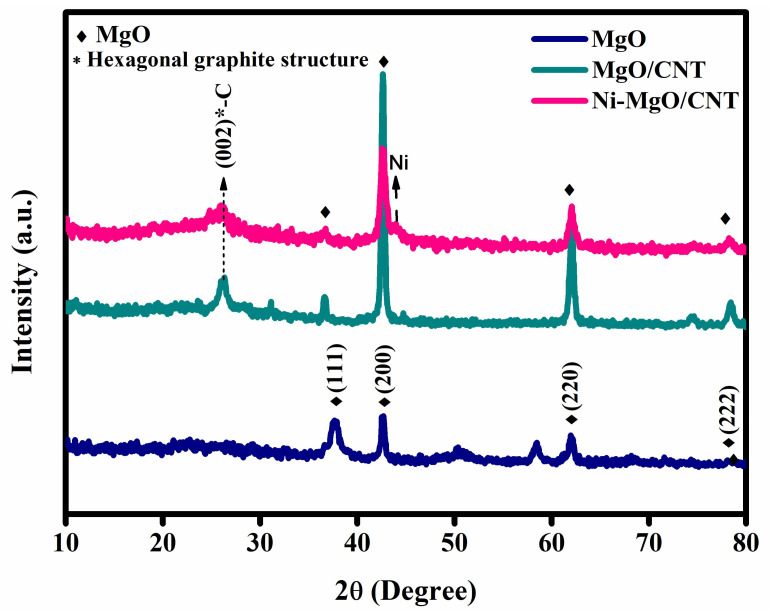
XRD analysis of MgO, MgO/CNT and Ni-MgO/CNT.

In order to explore the phase stabilities and nanostructures of the MgO, MgO/CNT and Ni-MgO/CNT, the constituent phases were explored using X-ray diffraction (XRD). Figure 1 exhibits the XRD peaks from the MgO (dark blue), MgO/CNT (green) and Ni-MgO/CNT (pink). All the characteristic peaks shown in the XRD pattern of MgO (dark blue) are related with the peaks from the (111), (200), (220) and (222) crystalline planes of MgO(standard JCPDS card no 75-1525),with the cubic crystal under an Fm-3m space group as explored in the earlier reports [29]. The high intensity and sharp peaks of the MgO/CNT demonstrated the prepared product’s good crystallinity. Also, the diffraction peaks at 25° to 26° indicate the presence of a carbon peak due to the typical multilayer nanotube structure [30,31]. No impurities or extra diffraction peaks were found with the CNT addition, which confirmed the phase purity of the material formation. The comparison of the peaks from the Ni-MgO/CNT to those from the MgO/CNT revealed no major differences except the broad and low intensity peak at 2θ = 44.2° in the Ni-MgO/CNT. No clear crystalline peaks were observed from the (111), (200) and (220) peaks from crystalline Ni [32]. The broad and low intense diffraction peak centered at around 44~45° is known to belong to the amorphous Ni (JCPDS card no 87-0712) [33,34]. It is interesting to note that no sharp peaks from Ni were observed in the Ni-MgO/CNT. The formation of amorphous Ni using nickel nitrate hexahydrate (Ni(NO_3_)_2_·6H_2_O) using the liquid-phase chemical reduction approach has been reported previously [35].

**Figure 2:**
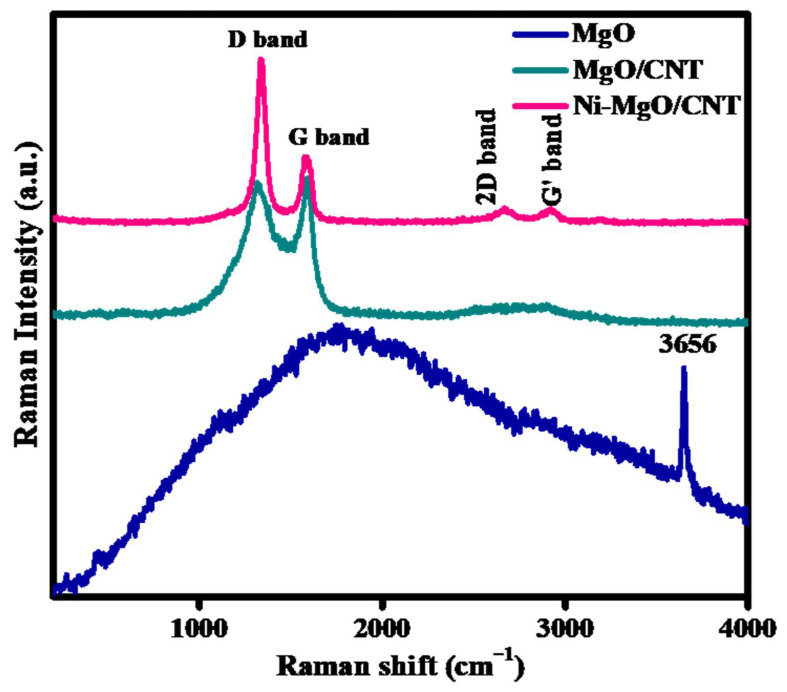
Raman spectra of MgO, MgO/CNT and Ni-MgO/CNT.

Figure 2 demonstrates the differences in the characteristic chemical structures of the as-prepared pristine MgO, MgO/CNT and Ni-MgO/CNT samples revealed using Raman studies. For pristine MgO (dark blue), a small peak observed at 442 cm^−1^ is linked with the presence of Mg, and the peak located at 3656 cm^−1^ indicates the vibrational broadening of the –OH group. The spectra ofthe MgO/CNT (green) and Ni-MgO/CNT (pink) in Figure 2 demonstrate the presence of two pronounced bands at 1333 (D-band) and 1591 cm^−1^ (G-band). The D-band is known to reveal defects or lattice distortions of carbon atoms, whereas the G-band is correlated with graphite E_2g_ and/or is brought about by sp^2^-bonded carbon atoms. The crystallinity of graphite or the CNT is supported by the G-band in Figure 2. The I_D_/I_G_ in Figure 2 was estimated with values of 2.11 and 0.97 for the Ni-MgO/CNT and MgO/CNT electrocatalyst, respectively. Fe, Co orNi are commonly employed as the catalyst for CNT synthesis [34,35,36,37,38]. The (I_D_/I_G_) relative intensity ratio is greater in the Ni-MgO/CNT, demonstrating defects in the synthesized Ni-MgO/CNT compared to those in the MgO/CNT. More defects might be introduced during the rapid growth of the CNT that are promoted by the catalysis reaction of Ni. Small peaks situated at 2676 and 2933 cm^−1^ were revealed as the second order of 2D and G′ bands. A small peak observed at 2933 cm^−1^ is known to be characteristic of the presence of CNTs. Here, the presence of a G′ band supports the higher fraction of CNTs in the Ni-MgO/CNT than in the MgO/CNT (green), although more defects were introduced in the CNT of the Ni-MgO/CNT [32,33,34,35,36] because of its faster growth facilitated by the catalytic function of Ni.

### 3.2. Microstructural/Nanostructural Analysis

**Figure 3:**
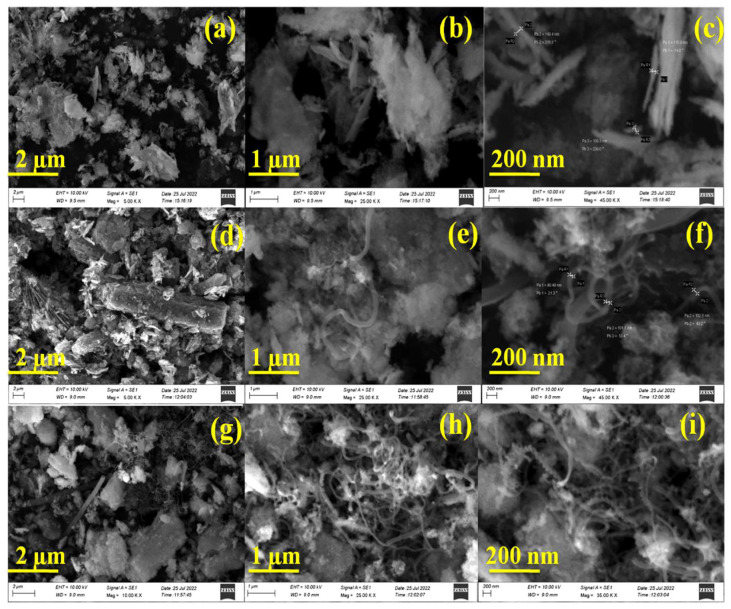
FE-SEMstudies: (**a**–**c**) MgO, (**d**–**f**) MgO/CNT and (**g**–**i**) Ni-MgO/CNT.

Figure 3 shows the surface morphologies of the as-synthesized MgO (a–c), MgO/CNT(d–f) and Ni-MgO/CNT (g–i) electrode materials observed using as canning electron microscope (SEM). Figure 3a shows the plate-like fuzzy appearance of the MgO sample [37,38,39]. The high magnification image in Figure 3b,c clearly exhibits the plate-shaped MgO crystals on the surface. The (100) plane of MgO is suggested to be stable because of its low surface energy [39], and the (110) plane is therefore naturally exposed after cleavage, and it is also the most favorably exposed surface generated by wet chemical methods as shown in the present study. The plate-shaped crystals are likely MgO crystals [40]. Figure 3d–f depicts the morphologies of CNTs grown on MgO using acetylene (C_2_H_2_) as a carbon precursor at 800 °C for 20 min of gas flow. The long and thin, curly tube-shaped CNTs are found to be formed over MgO and are synthesized from an acetylene (C_2_H_2_) precursor at 800 °C for 20 min of gas flow. The approximate thickness and length of CNTs formed on the surface of the MgO catalysts were observed to be ~100 nm and few micrometers, respectively. The appearance of the CNTs shown in Figure 3e,h is compatible with the CNTs observed by other investigators [30,31]. Figure 3g–i displays the well-aligned, helical-shaped CNTs on a flake-like structure of Ni-MgO, which clearly revealed the formation of a fair amount of curly CNTs on the surface, which was induced by the catalytic effect of Ni on the substrate surface. The abundance of CNTs in the Ni-MgO/CNT (Figure 3h,i) compared to those in the MgO/CNT (Figure 3e,f) supports the faster growth facilitated by the catalytic action. Furthermore, the CNT diameters could be decreased by adding a small amount of Ni content [41]. From the SEM images of the Ni-MgO/CNT (Figure 3h,i), the average diameter value was observed as 15–20 nm.

**Figure 4:**
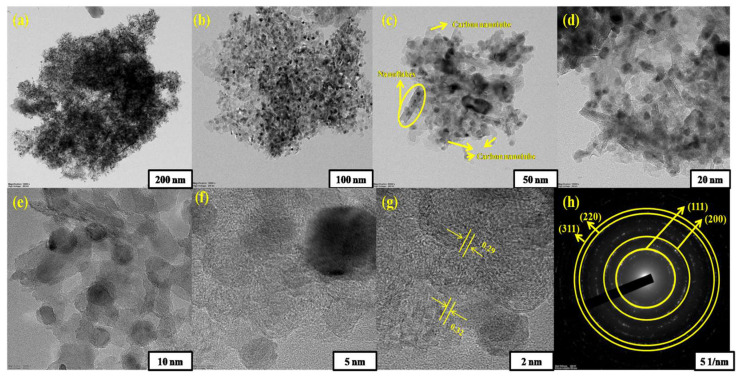
TEM studies: (**a**–**e**) Ni-MgO/CNT, (**f**,**g**) lattice fringes of Ni-MgO/CNT and (**h**) SAED pattern of Ni-MgO/CNT.

The nanostructure of the synthesized Ni-MgO/CNT nanocomposite was examined using the transmission electron microscopy (TEM) study, and its nanostructural details are illustrated in Figure 4a–h. The high-magnification TEM images presented in Figure 4a–e vividly exhibit the morphology of the nanoflakes encapsulated by conductive carbon nanotubes (CNTs), with the average diameter ranging from 15 to 25 nm. These observations align well with the findings from scanning electron microscopy (SEM) studies. Furthermore, Figure 4f,g distinctly revealed lattice fringes corresponding to MgO at the (111) and (200) planes, with interlayer distances of 0.29 and 0.32 nm, respectively, consistent with results obtained from the XRD analyses. The nanostructure of the Ni-MgO/CNT nanocomposite is further validated using selected area electron diffraction (SAED) and presented in Figure 4h, highlighting the lattice planes (111), (200), (220) and (222), corresponding well with the XRD results [42,43,44,45]. Additionally, the SEM images of the prepared Ni-MgO/CNT nanocomposite reveal an aggregated structure of the carbon nanotubes with a noticeable cracked curvature. This phenomenon is likely a result of covalent modifications occurring in the functionalization of the outer wall structural defects. Furthermore, the existence of defects within the nanostructure will diminish the toughness of the metal–carbon matrix. From the previous literature on metal oxide/CNTs, it is suggested that the synergistic effect between the metal and carbon components enhances electron transfer, thereby improving the overall efficiency [46,47]. The SEM/TEM images of the Ni-MgO/CNT nanocomposite reveal the initial phase of nanotube development. According to the earlier literature of Liu et al. [48], the metal integrated into the carbon network can serve as a catalyst for carbon graphitization in the vapor phase. As a result, the catalyst and carbon nanotubes have simultaneously overlapped with each other during the formation. Moreover, the interfacial adhesion occurring on the nanotube surface induces cracks and entanglement in the tube alignment, resulting in excessive separation during the nanotube formation [49].

**Figure 5:**
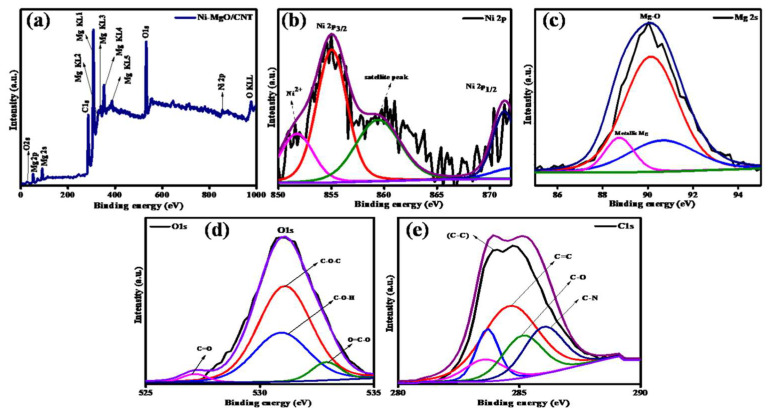
(**a**–**e**) XPS spectra of Ni-MgO/CNT; (**a**) overall survey spectra, (**b**) Ni2p, (**c**) Mg2s, (**d**) O1s and (**e**) C1s.

The surface elemental composition and chemical and binding energies were examined using X-ray photoelectron spectra (XPS). The successful formation of the Ni-MgO/CNT nanocomposite with the constituent elements of Ni, Mg, O and C is confirmed by the peaks from these constituent elements, as exhibited in Figure 5a. The core broad spectrum of Ni 2p at 855 eV explored Ni 2p_3/2_, and their satellite peaks observed at 860.3 eV denote the abundant presence of a Ni^2+^oxidation state. Additionally, a peak at 871 eV can be assigned to Ni 2p_1/2_in Figure 5b [50]. The typical peaks of Mg 2s located in the binding energy at 88.7, 90.0 and 91.3 eV can be attributed to those from the surface MgO layer and the chemisorbed surface compounds of Mg with carbon and oxygen such as MgOH and MgCO, as depicted in the Figure 5c [51], which matches the previous report of Hassan et al. [52]. The O1s spectra of oxygen positioned at 527.1, 531and 532.9 eV in Figure 5d correspond to the oxygen functional groups of C=O, C–O–C/C–O–H and O=C–O observed for the Ni-MgO/CNT, and are shown in Figure 5d [53]. The C1s spectra at 284, 284.6, 285.1 and 286.1 eV in Figure 5e of the prepared electrocatalyst represent the peaks associated with C–C, C=O, C–O and C–N [54].

### 3.3. Electrocatalytic Hydrogen Evolution Reaction

**Figure 6:**
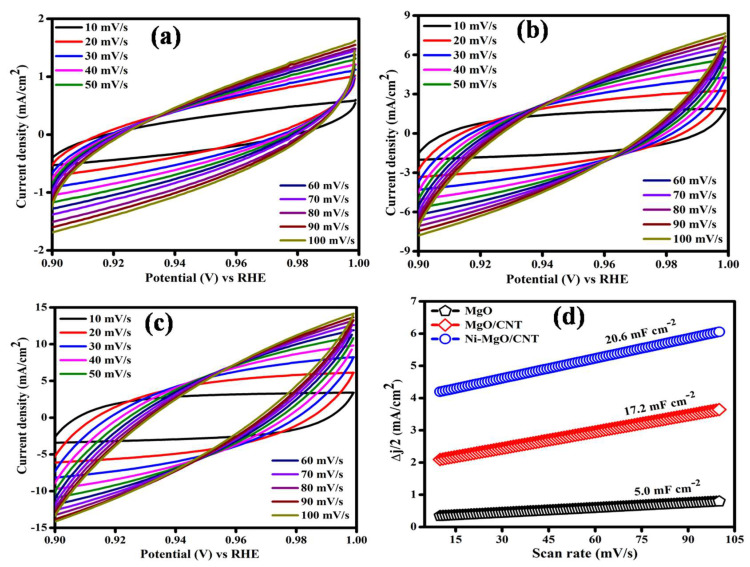
(**a**–**c**) CV curves; (**d**) ECSA linear plot of the prepared MgO, MgO/CNT and Ni-MgO/CNT nanocomposite.

The HERs were analyzed in three electrodes consisting of a working electrode (active material), reference electrode (Ag/AgCl) and counter electrode (graphite rod) using a 1 M alkaline KOH electrolyte solution in an ambient environment. The cyclic voltammetry curves of the prepared pure MgO, MgO/CNT and Ni-MgO/CNT were studied from 0.9 to 1.0 (V) vs. the RHE varying from 10 to 100 mV/s (Figure 6a–c).The attained CV exhibited an ideal electrochemical double-layer capacitance (EDLC) behavior and maintained a good charge storage. Additionally, the ECSA values were estimated by applying ECSA = C_dl_/C_s_, and are depicted in Figure 6d. The optimized Ni-MgO/CNT electrocatalyst achieved large C_dl_ and ECSA values of 20.6 mF cm^−2^ and 515 cm^2^, respectively, which are higher than those (5 mF cm^−2^ and 125 cm^2^) of the pure MgO and the values (17.2 mF cm^−2^ and 430 cm^2^) of the MgO/CNT. The large electrochemical surface area of the Ni-MgO/CNT appears to be responsible for creating a large reactive active site and escalating the possibility of subsequent ion charge transport [55]. Accordingly, the Ni-metal oxide-based electrocatalyst delivers a high catalytic activity due to the confinement effect [56].

**Figure 7:**
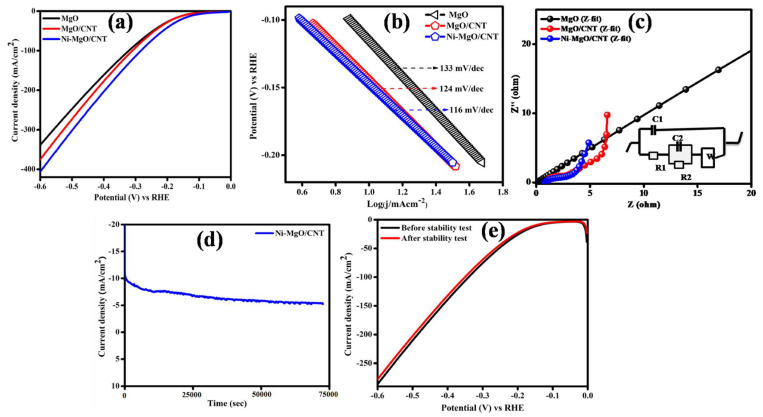
(**a**) LSV curve, (**b**) Tafel plot, (**c**) EIS (fitted) analysis, (**d**) chronoamperometry (CA) test and (**e**) LSV analysis after CA for the prepared MgO, MgO/CNT and Ni-MgO/CNT.

The linear sweep voltammetry comparison of the prepared pure MgO, MgO/CNT and Ni-MgO/CNT catalysts at 2 mV/s was explored and is presented in Figure 7a. Among all other samples, the Ni-MgO/CNT nanocomposite explored had the lowest value (117 mV) at10 mA/cm^2^, whereas the pure MgO and MgO/CNT exhibited the highest over potential values of 147 mV and 141 mV, respectively. This suggests that incorporating Ni as a host element into the MgO/CNT greatly improves the charge transfer efficiency and lowers the activation energy barrier to achieve an enhanced HER catalytic reaction. The pivotal enhancement of rigidity and robustness relies on the effective transfer of the load between the interfacial bonding of the metal oxide (Mg–O) and carbon nanotube (CNT) nanocomposite. As a result, the active participation of the metal oxide MgO is crucial for improving the electrocatalytic performance [57]. The enhanced electrochemical performance of the fabricated Ni-MgO/CNT electrocatalyst was compared with that which was reported in the earlier literature [58,59,60,61,62,63,64,65]. It will help to analyze the novel and integrated level of the electrocatalytic activity. For instance, the attained overpotential values were compared with the previous literature as expressed in Table 1.

Additionally, the intrinsic catalytic kinetics of the prepared samples as rate-limiting steps were evaluated using the Tafel slope analysis using ɳ = b log (j) + a, where ɳ denotes the overpotential, j is the current density and b denotes the Tafel slope, which is shown in Figure 7b.Accordingly, the Ni-MgO/CNT nanocomposite electrocatalyst achieved a 116 mV/dec Tafel value, smaller than the pure MgO (133 mV/dec) and the MgO/CNT (124 mV/dec).The hydrogen evolution reaction consists of semi-half reactions of electrochemical water splitting which involve the two-step electron transfer process. Initially, the molecular hydrogen from water was generated via an HER through three different reaction pathways named as Volmer, Heyrovsky and Tafel. In an alkaline medium, primarily the adsorbed hydrogen intermediates (H_ads_) were formed by the hydrolysis of the water molecule (H_2_O + e^−^ + catalyst → catalyst-H_ads_ + OH^−^), which we will call the Volmer step (discharging). Also, the adsorbed hydrogen unites with the H^+^ and e^−^ to form H_2_, which is known as the Heyrovsky step (desorption). The two adsorbed hydrogen on the catalyst surface combine to form an H_2_ molecule, acknowledged as the Tafel step (chemical desorption step) [66]. Hence, the achieved Tafel value of 116 mV/dec is lower than the Volmer step (120 mV/dec), indicating that the optimized electrode has a good agreement with the Volmer–Heyrovsky step of the reaction mechanism of the hydrogen absorption process (M-H_ads_) occurring on the electrode surface, which can increase the charge transfer rate of the Ni-MgO/CNT electrocatalyst [67].

Figure 7c shows the Nyquist plot of the as-prepared MgO, MgO/CNT and Ni-MgO/CNT samples. Comparatively, the Ni-MgO/CNT electrode exposed a lower solution and charge transfer resistance compared to the MgO/CNT and MgO electrodes because of the inverse correlation of the charge transfer reaction kinetics and minimum charge transfer resistance of the Ni-MgO/CNT electrode, which is obviously seen in the Nyquist plot with the clear evidence of a reduced arc. The electrochemical impedance (EIS) of the Ni-MgO/CNT electrocatalyst was fitted with the corresponding equivalent circuit using Nyquist plots (Figure 7c). The semicircle in the low frequency illustrated an electrode charge transfer resistance (R_ct_); the Ni-MgO/CNT attained a low R_ct_ value of 1.5 Ω and solution resistance (R_s_) value of 0.75 Ω, which are comparatively lower than all other electrodes of the pure MgO and MgO/CNT [68].

The durability and stability of the fabricated electrode was systematically measured using chronoamperometry testing, where the starting potential was set at 10 mA/cm^2^. The insightful results are illustrated in Figure 7d, showcasing the electrode’s commendable durability. Notably, the electrode exhibited a robust stability, retaining 72% of its performance over a prolonged duration of 20 h. While a slight decline in the current density was observed, this change is further corroborated in the linear sweep voltammetry (LSV) curve after stabilization, depicted in Figure 7e [69]. This observed stability positions the Ni-MgO/CNT composite as an optimal electrocatalyst for enduring energy applications. The minimal degradation over an extended period, coupled with the slight decline in current density, show its reliability and suitability for a sustained electrocatalytic activity. These findings highlight the Ni-MgO/CNT potential for long-term energy applications, attributing its performance to a high thermal capacity within alkaline media.

## 4. Conclusions

A Ni-MgO/CNT nanocomposite with animproved specific surface area was synthesized through the cost-effective impregnation and chemical vapor deposition methods. The comparison of the peaks from the Ni-MgO/CNT to those from the MgO/CNT revealed no major differences except for the broad and low intensity peak at 2θ = 44.2° in the Ni-MgO/CNT. The broad and low intense diffraction peak centered at around 44~45°is known to belong to the amorphous Ni. The (I_D_/I_G_) relative intensity ratio observed in the Raman spectra is greater in the Ni-MgO/CNT, indicating more defects in the synthesized Ni-MgO/CNT compared to those in the MgO/CNT. The presence of a G′ band in the Raman spectra supports the higher fraction of CNTs in the Ni-MgO/CNT than in the MgO/CNT (green), although more defects were introduced in the CNTs of the Ni-MgO/CNT because of its faster growth facilitated by the catalytic function of Ni. The evaluation of the morphological features of the Ni-MgO/CNT nanocomposite revealed the presence of helical-shaped CNTs adorning the surface of the Ni-MgO flakes, with the growth of the CNTs facilitated by the catalytic activity of Ni. The abundance of CNTs in the Ni-MgO/CNT compared to those in the MgO/CNT supports the faster growth facilitated by the catalytic action. Notably, the electrochemical performance of the Ni-MgO/CNT demonstrated a remarkably low value of 117 mV at 10 mA cm^−2^, signifying an exceptional catalytic activity toward the hydrogen evolution reaction (HER). The explored high electrochemical surface area (ECSA) value of 515 cm^2^ and low charge transfer resistance of 1.5 Ω are indicative of enhanced active metal sites and efficient ion transportation during the electrode–electrolyte interaction. Notably, the processed electrode in the present study exhibited a robust stability, retaining 72% of its performance over a prolonged duration of 20 h. While a slight decline in the current density was observed, this change is further corroborated in the linear sweep voltammetry (LSV) curve after stabilization. The observed stability position of the Ni-MgO/CNT composite is as an optimal electrocatalyst for enduring energy applications. The commendable catalytic action and the enhanced stability of the synthesized electrodes suggest the potential reusability of the Ni-MgO/CNT electrode for diverse applications.

## Figures and Tables

**Table 1:** Comparison of overpotential with the previous literature.

Catalyst	Electrolyte	j (mA/cm^2^)	ɳ (mV)	Ref.
Ni-N-C-250/Pt	KOH	10	400	[58]
Ni/NiFe_2_O_4_@PPy	KOH	10	127	[59]
Ni-Cu-Ti/CNTs-Ni	KOH	100	140	[60]
Ni/0.1CePO_4_catalyst	KOH	10	120	[61]
Ni-MSACs	KOH	10	270	[62]
NS-Ni/Mo_2_C@NF	KOH	100	151	[63]
Ni_3_S_2_@NiS-250/NF	KOH	10	129	[64]
CNS/PHS/CP	KOH	10	186	[65]
**Ni-MgO/CNT**	KOH	10	117	Present study

## Data Availability

Data are available on request.
